# Quality of life outcomes after transobturator tape full removal surgeries: A monocentric experience

**DOI:** 10.1002/bco2.317

**Published:** 2024-03-20

**Authors:** Marie‐Aimee Perrouin‐Verbe, D‐Carolina Ochoa, Rachel Skews, Mez Acharya, Antonin Prouza, Hashim Hashim

**Affiliations:** ^1^ Bristol Urological Institute Southmead Hospital Bristol UK; ^2^ Department of Trauma and Orthopaedic Surgery Southmead Hospital Bristol UK

**Keywords:** complications, mesh, quality of life, stress urinary incontinence, transobturator tape

## Abstract

**Objective:**

The objective of this study is to describe a standardised technique of full TOT removal with groin dissection and to report clinical improvement, satisfaction, safety and long‐term functional, quality of life (QoL) and sexual QoL outcomes.

**Materials and methods:**

A retrospective review enrolling all women who had full TOT removal, in a tertiary referral centre from May 2017 to November 2020. Functional outcomes, satisfaction and QoL were assessed using a bespoke composite questionnaire (UDI‐6, EQ‐5D‐5L and ICIQ‐S) with additional questions on sexual QoL. Secondary outcomes were post‐operative recurrent stress urinary incontinence (SUI) and complication rate according to the Clavien‐Dindo classification.

**Results:**

Full TOT removal using a vaginal approach and bilateral groin/para‐labial incisions was performed in 67 patients. Chronic pelvic pain was the main indication for mesh removal (51% of cases, *n* = 34). QoL questionnaires were answered by 43 patients. The satisfaction rate was high 86% (*n* = 37), and 81% (*n* = 35) of the patients considered the surgery successful. Seventy per cent (*n* = 30) of patients returned to having a sexual life after surgery. Recurrent SUI was reported in 32% (*n* = 14) of cases. The complication rate was 10% (7/67), all of them Clavien–Dindo ≤2.

**Conclusion:**

Despite a high rate of postoperative bothersome SUI, full TOT removal with bilateral groin dissection improves pain and QoL. It is associated with a high overall satisfaction rate and an acceptable rate of complications.

## 1 INTRODUCTION

Complications after mid‐urethral mesh sling (TVT/TOT) placement can be present in the short or long term, including extrusion, exposure, pain and/or de novo bothersome lower urinary tract symptoms (LUTSs).^1^, ^2^, ^3^ These complications may warrant partial or complete removal of the mesh,^2^ and most of the time, multiple surgeries are required before the mesh can be completely removed.^4^

Surgical removal procedures may differ according to the type of complication and/or the most bothersome symptom. Partial or full removal may be proposed, and if TOT insertion was the index surgery, it might include groin dissection.^5^, ^6^

Although previous reports documented the outcomes of tape removal, including TOT, consequences reported so far are pain improvement, recurrence of LUTSs and stress urinary incontinence (SUI). There is a scarcity of data regarding functional outcomes such as quality of life (QoL), satisfaction and sexual QoL after mesh removal.^7^, ^8^, ^9^, ^10^

Furthermore, the surgical technique of full/complete TOT removal, including bilateral groin dissection, is rarely reported in the literature, as it is seldom performed, even in cases reported as ‘total’ TOT removal.^9^, ^10^, ^11^

Therefore, our study aims to describe the surgical technique of full TOT removal with bilateral groin dissection and assess medium‐term functional outcomes, QoL, satisfaction and rate of recurrent SUI and complications.

## 2 MATERIALS AND METHODS

After institutional review board (IRB) approval, a retrospective quality improvement project was conducted on all women who had a synthetic TOT removal in our tertiary referral centre from May 2017 to November 2020.

Data were retrieved regarding patient demographics and past medical history, including age, body mass index, comorbidities and bladder drainage method/voiding mode. We also identified the date, surgical procedure and approach of the initial synthetic tape/mesh surgery, the brand, the type and the number of previous attempts to remove the tape.

Surgical indications were categorised as follows: erosion/extrusion/exposure (vaginal, bladder or urethra), voiding dysfunction, storage symptoms, recurrent UTI (defined as ≥3 episodes per year), chronic pelvic pain/dyspareunia (at least 2 months duration^6^) and mixed urinary symptoms/pain. Revision surgeries were classified into two groups: full removal or completion removal of their mid‐urethral transobturator tape.^12^ The length of tape removed was reported, as well as the presence of an orthopaedic surgeon with expertise in pelvic surgery.

### 2.1 Preoperative workup

The operative report of the initial surgery was requested, to understand better the procedure previously performed. All patients had a complete vaginal and pelvic floor examination. Further investigations (urethrocystoscopy, video‐urodynamic study, ultrasonography, pelvic MRI and CT scan) were performed according to the main complaint/symptoms and based on the diagnostic pathway previously published.^12^

The choice of the type of surgery and extent of tape removal was made via a joint shared decision process between the patient and the surgeon. All cases were discussed in a multidisciplinary meeting (including urologists who specialised in mesh/tape complications, urogynaecologists, nurse specialists, psychologists, radiologists, physiotherapists, pain specialists and orthopaedic surgeons).

Informed consent was obtained explaining the procedure its benefits and harms in detail. A patient decision aid for mesh removal developed by the National Institute for Health and Care Excellence (NICE)^13^ was given to patients.

### 2.2 Surgical technique

#### 2.2.1 Patient positioning

Standard lithotomy position with legs set in hydraulic boot stirrups with hip and knee flexion to 90° and slight external rotation of the foot. To gain more accessible access to the anterior vaginal wall, the patient is positioned in the Trendelenburg position.

#### 2.2.2 Vaginal approach

**FIGURE 1:**
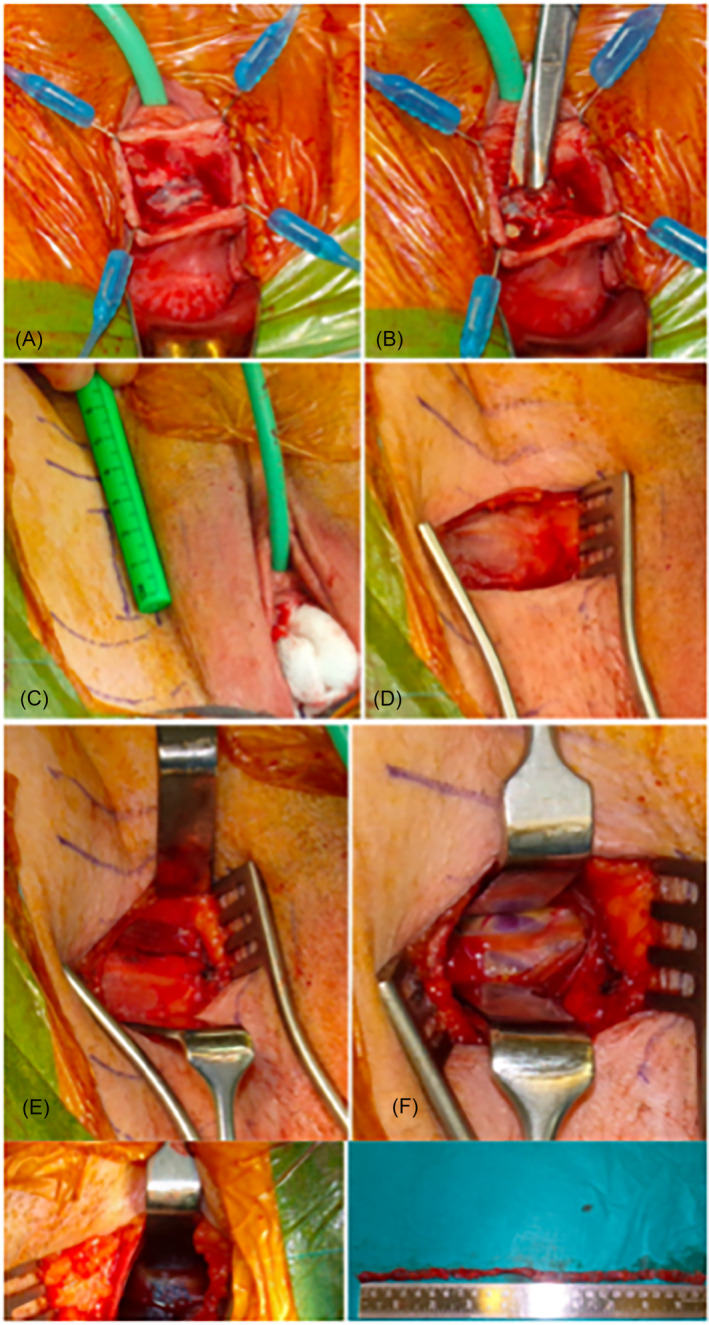
Step by step technique of full TOT removal with bilateral groin dissection. (A) Exposed vaginal portion of mesh. (B) Division of mesh laterally from urethra. (C) Paralabial incisions as a first step during grown dissection. (D) Intact fascia of the groin. (E) Horizontal incision of the fascia exposing the gracilis muscle fibres. (F) Muscle sparing technique of groin dissection. (G) Blue mesh identified deep with the groin. (H) Completely removed transobturator tape

A 2‐cm vertical sub‐urethral midline incision using a number 10 scalpel blade is made on the anterior vaginal wall. If the tape is felt more proximally, the position of the incision can be adjusted to provide appropriate exposure to the sling. Once the sub‐urethral part of the tape is exposed and visualised, a careful paraurethral dissection with fine curved dissecting scissors, such as McIndoe or Metzenbaum, is carried out (Figure 1A,B). We tend to divide the tape approximately 5 mm off the midline, thus reducing the risk of injuring the urethra. The tape is carefully dissected off the urethra, and dissection is continued on each side close to the tape, which is followed up to the obturator membrane bilaterally.

#### 2.2.3 Groin dissection

Groin dissection is commenced by a 3–5 cm vertical para‐labial incision in the skin crease from the origin of the adductor muscle tendon at the level of the clitoris (Figure 1C). Electrocautery is used to dissect the subcutaneous soft tissues until the superficial fascia of the thigh is reached (Figure 1D). The arm of the mesh is rarely identified above the fascia, which is incised horizontally using a blade. The underlying muscles are exposed (gracilis, adductor brevis and external obturator muscles) (Figure 1E). In contrast to other reported techniques in the literature, we avoid cutting the muscles to preserve their integrity by utilising sharp and blunt dissection to spread between the muscle fibres to access the obturator membrane and find the mesh^14^, ^15^ (Figure 1F). Surgeons must be aware of the obturator nerve and its branches and be careful not to damage it. Once the lateral aspect of the sling is found (Figure 1G), sling dissection to the outer surface of the obturator membrane is carried out. Every effort must be made to handle the mesh with caution to prevent it from snapping and thus risking a successful full removal. At this point, the whole mesh would be mobilised apart from the part incorporated in the obturator membrane, which is often adherent behind the bone. To remove the sling completely, a right‐angled clamp is introduced in an outside‐in fashion through the groin incision over the surgeon's finger following the lateral arm of the mesh. The instrument's tip is used to pierce the obturator membrane in front of the mesh. The instrument is partially opened within the membrane, which is usually sufficient to free the remaining adhesions and remove the mesh. The same technique is used to remove the mesh contralaterally. The mesh is therefore removed in two pieces. A marking nylon suture can be applied to both vaginal and groin ends of the mesh to ensure a full removal is achieved.

In some instances of urethral extrusion, a Martius labial fat pad interposition flap is needed. Concomitant autologous fascial sling (AFS) was sometimes proposed in patients with bothersome pre‐operative urodynamic SUI.

At the end of the surgery, photos of the tape are taken for the patient's chart (Figure 1H), and the mesh is sent for histology.

### 2.3 Postoperative assessment

**FIGURE 2:**
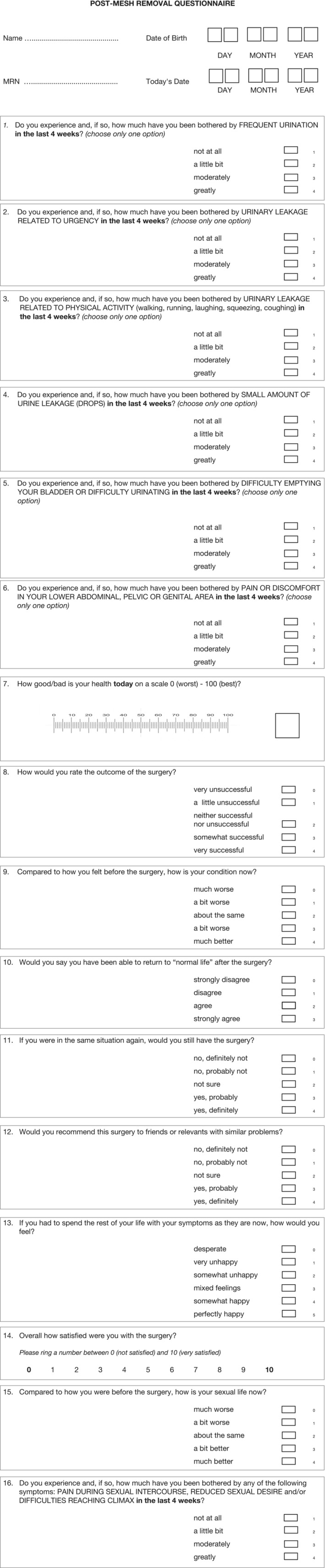
Post‐mesh removal questionnaire

Post‐operative complications were reported using the Clavien‐Dindo classification system.^16^ Post‐operative QoL and satisfaction were assessed using a bespoke composite questionnaire (UDI‐6, EQ‐5D‐5L and ICIQ‐S) (Figure 2) with additional questions on sexual QoL, which was undertaken by telephone or post/emails. Other functional outcomes (final symptom status: resolved, improved or unchanged/SUI recurrence), as well as late post‐operative complications, were also reported.

### 2.4 Statistical analyses

Demographic and clinical characteristics were reported using descriptive statistics, including median and frequencies. Categorical variables were expressed as frequencies and percentages. Quantitative variables were expressed as median (range min‐max).

## 3 RESULTS

During the study period, 67 patients had full TOT removal: full removal (*n* = 50) and completion (*n* = 17) with bilateral groin dissection. Forty‐three patients answered the QoL and satisfaction questionnaires.

**FIGURE 3:**
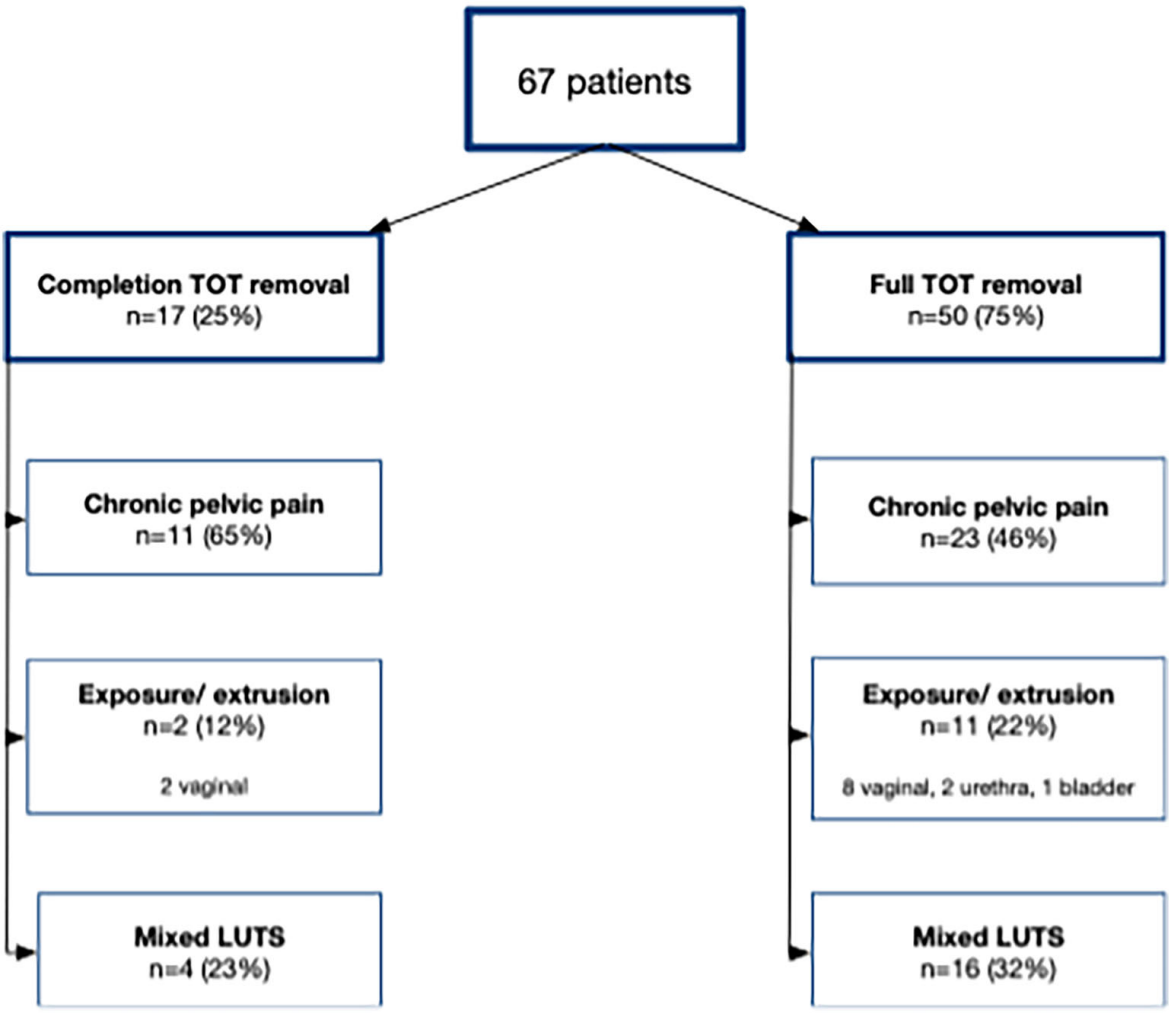
Indications for removal surgery

Patient's median age was 51 years (34–71), and their body mass index (BMI) was 33 (28–37). 9% (*n* = 6) had a history of two tape insertions. Twenty‐one per cent (*n* = 14) had previously undergone at least one tape removal and attended for complete removal for persistent pain and/or bothersome LUTS. The median interval between tape insertion and removal was eight years^1^, ^2^, ^3^, ^4^, ^5^, ^6^, ^7^, ^8^, ^9^, ^10^, ^11^, ^12^, ^13^, ^14^, ^15^ (Table 1). Chronic pelvic pain was the main indication for mesh removal in 51% (34/67), and mesh exposure/erosion in 19% (13/67) (*n* = 2 urethral, *n* = 10 vaginal and 1 bladder). Other indications were bothersome mixed LUTS in 30% (*n* = 20), mainly urgency or voiding dysfunction (Figure 3).

**TABLE 1:** Patient's characteristics

Variable	Total *n* = 67
Age, years (median, range)	51 (34–71)
BMI, *n* (%)	
<18.5	0
≥18.5 to <25	8 (12)
≥25 to <30	9 (13)
≥30	21 (31)
Unavailable	29 (44)
Number of patients with at least two tape insertions, *n* (%)	
Overall	
2 tapes:	6 (9)
TVT then TOT	3 (4.5)
TOT then TVT	3 (4.5)
TOT then TOT	0
3 tapes	0
History of mesh removal (*n* = 14, 21%)	
1 attempt (*n*, %)	8 (12)
2 attempts	2 (3)
≥3 attempts	4 (6)
Interval between initial tape insertion and removal, years (med, range)	8 (1–15)

The median surgical time was 180 min. An orthopaedic surgeon was present in 48% of surgeries (*n* = 32). The median length of mesh removed was 17.5 cm (11–26) for full removals and 15 cm (5–25) for full completions. Concomitant autologous fascial sling was performed in seven patients (10%), in whom bothersome urodynamically proven recurrent SUI was identified before mesh removal. Two patients (3%) had a concomitant Martius labial fat‐pad flap and urethral repair because of adherent tape to the urethra and per‐operative urethral adhesion from the mesh. Median post‐operative catheter length was ≤3 days, and hospital stay was ≤5 days. The complication rate was 10% (*n* = 7) with Clavien‐Dindo ≤2. The learning curve for the urologist to perform groin dissection independently was 15 cases.

### 3.1 Post‐operative outcomes

#### 3.1.1 Functional outcomes

Functional outcomes and QoL assessment were available for 43 patients (64%), median follow‐up 32 months (12–54). Among the 34 patients who underwent mesh removal for chronic pelvic pain, 27 answered the questionnaire, and 52% (*n* = 14) reported pain improvement (≤2 on Question 6 UDI‐6).

Among the 18 patients with pre‐operative voiding dysfunction, 13 answered the questionnaire, and 85% (*n* = 11) reported an improvement (≤2 on question 5 UDI‐6).

Among the 17 patients with pre‐operative urgency/urgency urinary incontinence, only eight patients answered the questionnaire, with 37.5% (*n* = 3) reporting an improvement (≤2 on questions 1, 2 UDI‐6).

Twenty patients described pre‐operative SUI, and 62% (*n* = 10) remain incontinent. However, among the nine patients who had concomitant AFS, seven answered the questionnaires, and 86% (*n* = 6) of them improved (≤2 on Questions 3 and 4 UDI‐6). In the overall population, recurrent (de novo) SUI was reported in 32% (*n* = 14) of cases, and overall post‐operative bothersome urgency was reported in 37% of the patients (*n* = 16).

Satisfaction rate was high 86% (*n* = 37); 81% (*n* = 35) considered the surgery successful, 73% (*n* = 33) felt better or much better, 93% (*n* = 40) would still have the surgery if they were in the same situation, and 95% would recommend this surgery.

In the group of patients who answered the questionnaire, 70% (*n* = 30/43) returned to having a sexual life after surgery 80% (*n* = 24/43) consider it at least the same as before. Thirty per cent (*n* = 18) experienced post‐operative sexual dysfunction, such as pain during intercourse, reduced sexual desire or difficulties reaching climax (Table 2).

**TABLE 2:** Quality of life outcomes

Questionnaire	*N* = 43 patients
**UDI‐6** For each question, circle the number that best describes this problem for you over the past month. 0 = *not at all*, 1 = *a little bit*, 2 = *moderately*, 3 = *greatly* Do you experience and, if so, how much are you bothered by:	*N* with score = 3 (%)
1 Frequent urination?	16 (37%)
2 Urine leakage related to urgency?	16 (37%)
3 Urine leakage related to physical activity?	14 (32%)
4 Small amount of leakage?	15 (35%)
5 Difficulty emptying your bladder or difficulty urinating?	5 (12%)
6 Pain or discomfort in your lower abdominal, pelvic or genital area?	16 (37%)
UDI 6 score (median, IQR)	4 (12)
EQ‐5D‐5L How bad/good is your heath today on a scale between 0 (worst) and 100 (best) Score (median, IQR)	67.5 (30)
**ICIQ‐S**	
7 How would you rate the outcomes of the surgery? 0 = *very unsuccessful*, 1 = *a little successful*, 2 = *neither successful, nor unsuccessful*, 3 = *somewhat successful*, 3 = *very successful*	35 (81%) with score ≥3
8 Compared to how you felt before the surgery, how is your condition now? 0 = *much worse*, 1 = *a bit worse*, 2 = *about the same*, 3 = *a bit better*, 4 = *much better*	33 (73%) with score ≥3
9 Would you say you have been able to return to normal life after the surgery? 0 = *strongly disagree*, 1 = *disagree*, 2 = *agree*, 3 = *strongly agree*	18 (42%) with score ≥2
10 If you were in the same situation again, would you still have the surgery? 0 = *no, definitely not*, 1 = *no, probably not*, 2 = *not sure*, 3 = *yes, probably*, 4 = *yes, definitely*	40 (93%) with score ≥3
11 Would you recommend this surgery to friends or relatives with similar problems? 0 = *no, definitely not*, 1 = *no, probably not*, 2 = *not sure*, 3 = *yes, probably*, 4 = *yes, definitely*	41 (95%) with score ≥3
12 If you had to spend the rest of your life with your symptoms as they are now, how would you feel? 0 = *desperate*, 1 = *very unhappy*, 2 = *somewhat unhappy*, 3 = *mixed feelings*, 4 = *somewhat happy*, 5 = *perfectly happy*	10 (23%) with score ≥4
Sum Score Score (median, IQR)	12 (19)
13 Overall how satisfied were you with the surgery? (*not satisfied*) 0–10 (*very satisfied*)	37 (86%) with score ≥5
14 Compared to how you were before the surgery, how is your sexual life? 0 = *much worse*, 1 = *a bit worse*, 2 = *about the same*, 3 = *a bit better*, 4 = *much better*	24 (80%) with score ≥2
15 Do you experience any symptoms related to sexual dysfunction, such as pain during intercourse, reduced sexual desire or difficulties reaching climax? 0 = *not at all*, 1 = *a little bit*, 2 = *moderately*, 3 = *greatly*	18 (30%) with score ≥2

*Note*: Data regarding quality of life were available for 43 patients, with a median follow‐up of 32 months (12–54). For Questions 14 and 15, 70% of the patients (30/43) answered as they were sexually active post‐operatively.

## 4 DISCUSSION

To our knowledge, the present series has one of the highest numbers of full/completion TOT removals with tissue‐sparing groin dissection (*n* = 61). It is one of the few to report QoL, satisfaction and sexual outcomes. The surgery satisfaction rate was high (86%), with most patients considering it successful (81%), even if they were experiencing bothersome LUTS, for example, recurrent SUI. Moreover, 70% of our patients returned to sexual life, with 80% considering at least the same, even though one‐third experienced sexual dysfunction related to pain during intercourse or reduced desire.

In our series, the main indication for full removal/completion of TOT removal was chronic pelvic pain 51% (34/67). Current evidence states that pain without underlying causes, such as exposure or erosion, is responsible for 1–17% of sling removals.^17^ The surgical approach for treating pain differs among publications. Some authors recommend removing most of the material to decrease the source of the pain,^11^ mainly if the pain is unrelated to a specific component of the tape.^12^ Others advocate a partial removal. Leonard et al.^6^ suggest performing groin incision only in patients with preoperative obturator neuralgia to remove the prosthetic material and obturator nerve release. In patients with myofascial pain, it has been reported that a section of the material to release tension, without complete removal, may be sufficient.^18^

The amount of mesh removed and the pain response vary among publications. In 2014, Hou et al.^11^ reported a series of mesh removal for pain, including 54 tapes (38% 21/54 TOT). TOT removal with groin dissection was performed. Pain‐free status was achieved by 81% of their population, whatever the type of tape.^11^ In a more recent series, Mengerink et al.^17^ found no statistically significant difference in partial vaginal versus complete vaginal removal (*p* = 0.38). However, just one patient with groin dissection was included.

Even if these results may favour partial removal in cases of pain, one‐step full removal should be considered, as partial removals make future removals difficult due to retraction of the mesh.^12^ We advocate for full removal, including groin dissection, especially in patients with pelvic/vaginal pain. In one series, 84% of the patients with pain required more than one surgery for removal.^17^ In the current study, patients with chronic pelvic pain underwent full total/completion removal with groin dissection in 91% of cases (61/67). Leading to a high satisfaction and pain with low morbidity.

Complete TOT mesh removal may be challenging as it requires groin dissection, and surgeons and patients may be aware of the possible complications.^12^ Despite that possibility, in our series, morbidity related to groin dissection was low; only two patients presented with wound infection without needing reoperation. Therefore, we consider that groin dissection, with appropriate training, could be offered to patients with mesh complications without significantly increasing morbidity related to the surgical procedure.

Surgical technique may differ for vaginal exposure only, where a partial excision of the tape with the removal of the sub‐urethral portion may be sufficient.^12^ However, we consider that the entire tape may be colonised,^12^ and full completion/removal was offered to our patients. Regarding voiding dysfunction, in our series, 85% of improvement was reported, consistent with the evidence 82%.^19^

In the current series, the rate of recurrent SUI was 32% consistent with the literature.^20^ However, it is lower when compared with other series of complete removal, in which up to 69% have been reported.^19^, ^21^ Indeed, a recent systematic review concluded that the risk of post‐operative SUI is lower after partial mesh removal compared to total (odds ratio 0.46, 95% CI 0.22–0.96).^22^ This must be balanced with the potential risk of the initial symptom persistence in case of partial removal. Post‐operative bothersome urgency was reported in 37% of the patients, which differs significantly from a series that reported an increase in urgency after total mesh removal in 80%.^21^

Our study has several strengths, including QoL assessment along with sexual life and satisfaction, and homogeneity of the groin incision technique, with reduced morbidity. The main limitation is its retrospective nature. Therefore, questionnaires were only completed post‐operatively. Also, only 64% of the overall population answered the questionnaires. Being a tertiary referral centre, and therefore having an unknown denominator of the total number of tape insertions, we could not calculate the incidence of tape complications; this limitation was also shared by different authors^9^, ^23^

Finally, the type and brand of tapes implanted were not always reported and from anecdotal experience, some of the meshes seem to cause more local inflammatory reaction than others but there are no data to available as to whether this translates to more complications.

## 5 CONCLUSION

TOT mesh removal with bilateral groin dissection improves patients' pain and QoL. It is associated with a high rate of overall satisfaction, low morbidity and an acceptable rate of SUI recurrence compared to partial removal, and less than that quoted in other series. Groin dissection should be in the armamentarium available for patients suffering with mesh complications.

## AUTHOR CONTRIBUTIONS

Marie Aimee Perrouin‐Verbe, Carolina Ochoa and Hashim Hashim contributed to the design. Marie Aimee Perrouin‐Verbe and Antonin Prouza contributed to data collection. Marie Aimee Perrouin‐Verbe, Carolina Ochoa, Rachel Skews and Mez Acharya contributed to the analysis of the results. Marie Aimee Perrouin‐Verbe and Carolina Ochoa contributed to writing the manuscript. Hashim Hashim designed the original and supervised the project.

## CONFLICT OF INTEREST STATEMENT

The authors declare that they have NO affiliations with or involvement in any organisation or entity with any financial interest in the subject matter or materials discussed in this manuscript.
